# Measuring Population Health from a Broader Perspective: Assessing the My Quality of Life Questionnaire

**DOI:** 10.5334/ijic.3967

**Published:** 2019-05-13

**Authors:** Roy J.P. Hendrikx, Hanneke W. Drewes, Marieke D. Spreeuwenberg, Dirk Ruwaard, Martine Huuksloot, Corine Zijderveld, Caroline A. Baan

**Affiliations:** 1 Tilburg University, Tilburg School of Social and Behavioral Sciences, Tranzo Scientific Center for Care and Welfare, NL; 2 National Institute for Public Health and the Environment, Center for Nutrition, Prevention and Health Services, Department for Quality of Care and Health Economics, NL; 3 Maastricht University, Faculty of Health, Medicine and Life Sciences, CAPHRI School for Public Health and Primary Care, Department of Health Services Research, NL; 4 Zuyd University of Applied Sciences, Research Centre for Technology in Care, NL; 5 Dutch Patient Federation, Orteliuslaan, NL

**Keywords:** population health, positive health, Triple Aim, evaluation

## Abstract

**Introduction:**

Population health perspectives increasingly focus on people’s perception of resilience, ability to adapt and self-manage. The goal of this study is to determine whether the *MijnKwaliteitVanLeven.nl* (“MyQualityOfLife.nl”) survey is a valid and reliable instrument for assessing the broader health perspectives at population level.

**Methods:**

19,809 entries of the MyQualityOfLife.nl survey were used. To assess face validity, Huber’s six dimensions of positive health were used as a framework for expert feedback. A confirmative factor analyses was done using the expert’s item clustering, followed by data-driven explorative factor analyses and reliability tests.

**Results:**

Experts distributed 74 of the 118 items over all six dimensions of positive health. The confirmatory factor analysis model based on expert classification was not confirmed. The subsequent exploratory factor analysis excluded most items based on factor loading and suggested two factors; ‘quality of life’ and ‘daily functioning’, both showing excellent reliability.

**Conclusion:**

The MyQualityOfLife.nl survey can assess the broader concept of health in a population as well as ‘quality of life’ and ‘daily functioning’. However, the survey can currently not evaluate several of the positive health dimensions separately. Further research is needed to determine whether this is due to the instrument or the positive health dimensions.

## Introduction

The evaluation of population health has become ever more important as integrated care reforms often use policies focused on entire populations [1]. Population (health) management (PM) initiatives, such as the American accountable health communities [2], Dutch pioneer sites [3] and English Vanguards [4], aim to improve the health of their population as well as the quality of services and affordability of their health systems (Triple Aim) [5]. In order to achieve these goals and have an impact on population health, they aim to integrate service across healthcare, including social care, and ideally try to bridge gaps that go outside healthcare [6,7]. These new integrated care policies have to be evaluated to determine if they have the desired impact and therefore instruments are needed that adjust for the population focus as well as the concept of population health.

Population health is generally defined “as the health outcomes of a group of individuals, including the distribution of such outcomes within the group [8].” What is considered ‘health’ here, however, has seen continuous discussion over the years [9]. The still dominant definition was created by the World Health Organization in 1948; “health is a state of complete physical, mental and social well-being and not merely the absence of disease or infirmity [10].” This definition has been highly debated since its inception. Recent changes in population characteristics and healthcare policy changed the focus towards a wider view on health [11,12,13,14,15,16]. There is a growing elderly population that could be considered sick in a traditional sense, as they have one or more conditions. However, many are able to live their lives independently. This led to a bigger emphasis on what people are still able to do and empowering them, focusing more on their abilities then limitations. Terms often brought up now in relation to health are people’s own perception of resilience, meaningfulness and ability to adapt and self-manage. “Conventional” dimensions, such as physical, mental and social health, are still considered relevant, but an additional emphasis is put on these new concepts [11,12]. Even though these concepts are not entirely new, as Canguilhem described the ‘ability to adapt’ back in 1943 [17], many see this broader approach as the way forward.

Most instruments have not adapted a broader perspective on health, even though the new dimensions are embraced in many countries’ healthcare reforms [18]. For example, the Short Form 12 only produces a physical and mental component score [19] and does not cover meaningfulness or social dimensions, which are also lacking in the EuroQol 5D [20]. Additionally, most instruments focus on individuals and are not meant to evaluate regions. This creates difficulties for PM initiatives, such as the Dutch pioneer sites, aiming to capture the full spectrum of population health, as they have limited instruments available to them to evaluate their interventions. Huber et al. (2016) suggested a quantitative operationalization of their dimensions of positive health, however this instrument has not yet been validated [21]. A potential instrument that could fill the gap is the existing *MijnKwaliteitVanLeven.nl* (“MyQualityOfLife.nl”) survey. This survey was created as a response to changes in the Netherlands in long-term care, which put a bigger emphasis on self-reliance. The goal of the survey was two-fold; 1) to monitor the health impact of the Dutch transition from long-term care being organised nationally to being predominantly organised by municipalities and 2) provide a conversation tool for individuals that helps them determine what matters to them regarding health. Topics covered in this predominantly online survey include experienced health, personal situation, abilities and limitations [22]. The survey is also widely used in the Netherlands, which, combined with its broad health topics, means that the survey could potentially provide a one-stop survey for PM initiatives.

There is a lack of instruments that can be used to evaluate the new broader concept of health in populations. The MyQualityOfLife.nl survey has yet to be studied as a population health instrument for use by PM initiatives. Aiming to fill this gap, this study therefore aims to determine whether the survey is a valid and reliable instrument for assessing the broader concepts of health at population level.

## Method

### Data collection

The Dutch MyQualityOfLife.nl instrument is a publicly available online survey that can be completed by anyone above the age of seventeen. Participation is stimulated using advertorials, pamphlets, and by general practitioners (e.g. during consultations) and hospitals. In addition, the Dutch Patient Federation sends volunteers to different care locations (e.g. nursing homes) to encourage and help patients fill out the survey in person. The initial target population of the survey differed from the current one. Starting in November 2014, three specific populations were targeted: Dutch adults over the age of 65, people with chronic conditions and (informal) caregivers. These populations were sought out as the survey was created to assess how people deal with the long-term care reforms in the Netherlands. However, the aim as well as the target population has expanded over the years. Everyone thinking about a healthy lifestyle is currently invited to complete the survey.

To partake, participants go to www.mijnkwaliteitvanleven.nl and fill out an application form. Once participants have applied, they receive an email with a web link to fill out either the long or the short version of the survey. Once they filled out the survey at least once and have agreed to a follow-up, they receive two yearly email invitations to fill out the survey again. For this study, only initial entries of the long version are used.

### Instrument

The survey, created and developed by the Dutch Patient Federation in 2014, consists out of 118 items classified by topic covering the participants’ views regarding their own health from a broader perspective and their experiences regarding the care and support they receive. These topics were determined and refined using focus groups, which included groups of different age categories, chronically ill patients and informal caregivers. Based on existing instruments, predominantly the Impact on Participation and Autonomy survey [23], questions were selected for each topic as described by the focus groups. Items were multiple choice, ratings, statements or open-ended. The final survey was tested using the think aloud protocol; participants were asked to fill out the survey and verbalize whatever crosses their mind [24]. This yielded several improvements regarding the usability of the survey; it especially led to a more logical order of questions.

### Positive health

As described above, a broader perspective of health is becoming more dominant [11,12,13,14,15,16]. What most new approaches have in common is a focus on a person’s own perception of health, resilience and abilities and they include dimensions beyond the physical, mental and social. The Meikirch Model of Health, for example, states that: health is a state of wellbeing emergent from conducive interactions between individuals’ potentials, life’s demands, and social and environmental determinants[15]. Walburg defines positive health as the ability to flourish and develop and to prevent disruptions such as diseases [12]. Acknowledging that there are multiple approaches, for this study Huber et al.’s (2011) concept of positive health, “health as the ability to adapt and to self-manage”, was used. It operationalizes the concept in six dimensions that can be used in evaluations: physical functioning, mental health, meaningfulness, quality of life, social participation and daily functioning (Appendix 1) [11].

### Analyses

First, face validity was tested using an expert panel. The panel existed out of six researchers familiar with the concept of positive health. Each filled out an online survey that included all items of the MyQualityOfLife.nl survey. They also received an infographic defining the six dimensions of positive health (Appendix 1). For each item, experts selected one of seven options, either one of the six dimensions of positive health or ‘not applicable’, if the item was considered not relevant for any dimension. Responses from this round were collected and combined into a single Excel file. This file was anonymised and showed the responses of each expert side-by-side. The experts were send the Excel file for a second round, in which they were asked to revaluate their own responses knowing the responses of other experts. The input from this round was used to then classify items. Any items that had three or more experts agreeing were classified as that positive health dimension or “not applicable”. If less than three experts agreed the item was classified as having “no consensus”. Second, the distribution of each item was used to check for floor and/or ceiling effects. Any item having more than 40% of responses at either end of the scale was considered to have a floor/ceiling effect. These indicators provide useful information when selecting items for relative evaluations, but for this study items showing a floor/ceiling effect were not excluded from further analyses.

Third, in order to assess whether the classification by experts could be found in the data, a confirmatory factor analyses was performed based on their input. Items that had not reached consensus were excluded from the confirmatory factor analysis. Fit was assessed using the chi-squared test, normed fit index, comparative fit index and root mean square error of approximation, focusing on the chi-squared test. Subsequently, to determine which factors the data itself would provide, a data-driven exploratory factor analysis was performed based on principal component scores with a direct oblimin rotation. Items that were considered not applicable were excluded and items that had no consensus were included, as they were overall considered potentially relevant. Sampling adequacy was established using the Kaiser-Meyer-Olking measure. Factor extraction was done using a scree plot by establishing the point of inflexion [25]. Factor patterns and item trimming was done based on factor loadings. To create clear factors each item had to have the highest factor loading above 0.6, while the second highest had to be below 0.4 [26].

Fourth, reliability was assessed per factor as identified by the factor analyses using Cronbach’s alpha [27]. This statistic was also used to asses which items could be excluded in a stepwise manner from the survey when measuring positive health. Additionally, corrected item-total correlations were used to assess convergent validity [28].

All quantitative analyses described above were performed using SPSS 22 (SPSS Inc., Chicago, Illinois) and R Studio Version 0.99.441 for Windows (RStudio, Boston, Massachusetts).

## Results

### Description of study population

Between November 2014 and February 2017, 19,809 participants filled out survey. The study population consisted mainly out of women, people born in the Netherlands and people with a chronic condition (Table 1). Compared to the Dutch population, the study population was older, more likely to be unemployed and/or have a chronic condition.

**Table 1:** Description of study population.

Population	Study	Dutch*

Total N	19,809	16,979,120
Gender (% male)	41.0	49.2
Age (Mean – SD)	59.1–14.3	41.5 – NA
65+ (%)	39.8	18.2
Education (% higher educated**)	33.8	23.6
Country of birth (% Netherlands)	94.0	77.9
Chronic condition (%)	76.1	30.3
Employed (%)	30.7	63.2

* Based on CBS (Statistics Netherlands) data [29,30,31,32], SD = Standard Deviation, NA = Not Available. ** Completed higher education i.e. Bachelor’s degree or higher.

### Face validity

**Figure 1:**
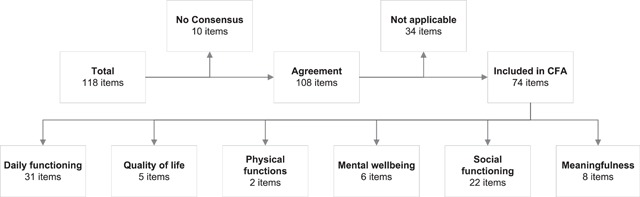
Flowchart of expert panel.

After two rounds of expert feedback, 108 out of 118 items had 3 or more or more experts agreeing, of which 65 were unanimous. No consensus was reached for ten items, which were excluded from the confirmatory factor analysis. For example, the statement “I spend time and attention on the things I feel are important” was split between the meaningfulness and mental health dimensions. Of the 108 items that reached consensus, 34 items were considered not applicable for assessing positive health; most of which were related to care use and the use of daily aids (e.g. “What is going well with your current aids?”). This meant that in total 44 of the 118 items that either reached no consensus or were considered not applicable, were excluded from the confirmatory factor analysis. The other 74 items that reached consensus and were considered relevant, covered all dimensions according to the experts, with most items (31) assigned to daily functioning (e.g. “Go wherever I want in my home is…”) and the least (2) to physical functioning (e.g. “I feel fit enough to do whatever I want”). Figure 1 illustrates the complete face validity process and the extensive results from both rounds of expert feedback can be seen in Additional File 1.

### Response analyses

As participants could not skip any items, there were no missing values in the data. Even though almost all responses were skewed positively, most did not reach the threshold of 40% to establish a ceiling effect (Appendix 2). Most had extremes receiving around 25–30% of responses, while others went far below that. The fourteen items that did, such as the statement “Going to bed and getting out of bed when I want goes…”, were predominantly related to personal care, social contact and mobility.

### Factor analyses

The 34 items considered not applicable by experts, were excluded from both the confirmatory and the exploratory factor analyses.

The classification into dimensions provided by the experts was used as a basis for the confirmatory factor analysis. Goodness-of-fit indices unanimously showed a bad fit (Table 2), indicating that the expected model did not seem to come forward in the data. When the data was stratified by age (over and under 65 years old), education (low/high) or chronic conditions (yes/no) this did not change.

**Table 2:** Results of Factor Analyses.

Confirmatory factor analysis	

Chi-squared/df	75,706*/2612
NFI	0.644
CFI	0.652
RMSEA	0.082*
**Exploratory factor analysis**	

KMO	0.966

* p-value < 0.001, df = degrees of freedom, NFI = Normed Fit Index, CFI = Comparative Fit Analyses, RMSEA = Root Mean Square Error of Approximation, KMO = Kaiser-Meyer-Olkin.

**Figure 2:**
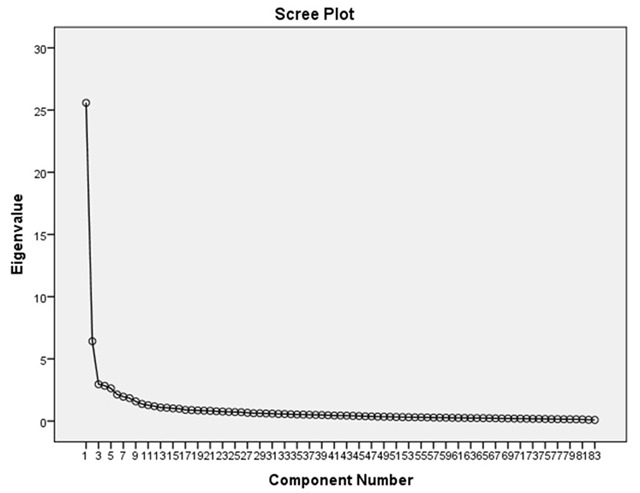
Scree plot of exploratory factor analyses.

Based on the Kaiser-Meyer-Olkin-test (Table 2), sample adequacy was satisfactory for an exploratory factor analysis. By assessing the scree plot (Figure 2), which did not change when correcting for the above mentioned demographic factors, two factors were selected. Based on the factor loading requirements, 50 items were excluded from either factor (Appendix 3). Of the two factors, factor two had the clearest theme as they were all related to ‘daily functioning’. Factor 1 consisted out of 24 items that emphasised ‘quality of life’ with items such as “I enjoy life” showing the strongest factor loads (0.806). However, mental well-being (“I can cope with change and setbacks”), meaningfulness (“My trust in the future when I think about my life”) and social participation (“I can rely on others if I need support or help”) were represented as well, but to a lesser degree.

### Reliability

The reliability for both factors found in the exploratory factor analysis was excellent (Cronbach’s alpha: factor 1 = 0.935, factor 2 = 0.900). By removing one item from factor 1, leading to 23 items, and a single item from factor 2, leading to seven items, some small improvement could be made (Appendix 4). Convergent validity was strong in both factors; both showed items with item-total correlations above 0.5 (Appendix 4).

## Discussion

To evaluate integrated care policies aiming to go beyond the individual (patient), instruments are needed that assess the broader concept of health at the population level. Instruments that measure people’s own perception of resilience, meaningfulness and ability to adapt to changes in their personal situation. The goal of this study was to determine whether the already available MyQualityOfLife.nl survey is a usable instrument to evaluate this broader perspective on population health. This study showed that face validity based on expert feedback was good as items covered all six dimensions of positive health, response dispersions were adequate and reliability of the found factors was high. Factor analyses, however, showed that only the broad ‘quality of life’ and ‘daily functioning’ dimensions could be extracted, while the others could not be individually distinguished. Thus, the MyQualityOfLife.nl survey can be used to assess a population’s overall health from a broader perspective, but it is unable to identify various specific dimensions, despite the apparent presence of items for each of them.

The survey shows promise as a comprehensive positive health instrument. The selected 74 items seem to cover all aspects belonging to the dimensions of positive health according to the experts and quantitatively they performed well. Additionally, many items were adopted from the Impact on Participation and Autonomy survey, which is a validated instrument [33,34]. These items have now proven to keep these good properties when used in the survey at population level, increasing their usability. These quantitative characteristics, combined with the collected expert input, suggest that items show promise for measuring specific aspects of each positive health dimension.

When the goal is to assess specific dimensions of positive health, the MKLV survey seemed to fall short. Results suggest that the MyQualityOfLife.nl survey is able to distinguish two out of six dimensions of positive health (daily functioning and quality of life) at population level. Explanations for this discrepancy could come from two sides. First, the survey was not specifically developed for the purposes tested in this study, which could have led to questions that were not divisive enough. It could be necessary to use more targeted surveys when the goal is, for example, to evaluate physical or mental health (e.g. Short Form 12) specifically. Multiple targeted health surveys have proven their use at the population level [35,36]. However, they still fall short when the goal is to cover the broader population health concept. They cannot be used as all-in-one solutions and lack the capability to assess recent additions to the concept of health. The second explanation may be that the dimensions of positive health are themselves not distinct enough and thus difficult to evaluate separately in a quantitative manner. A clear culprit from this perspective could be the strong correlation found between different dimensions. This cohesion could prevent the expected factor structure from coming forward and instead illustrating a single factor, or two, as was the case here. This suggests that if participants fill out a survey by themselves, they do not consciously differentiate between multiple dimensions of positive health. This is in contrast with when patients are specifically asked about the positive health concept, as they then do deem each dimension relevant [21].

Several aspects have to be considered when interpreting the results. The characteristics of the study population have to be taken into account. More elderly, chronically ill and highly educated participants were included in this study when compared to the Dutch population [33,34]. However, stratified factor analyses suggest that these differences should not have affected results. Furthermore, even though only a few questions showed a floor/ceiling effect, most were still positively skewed. This could have reduced possible dimension separation, an effect that will only be amplified in a more general and healthier population. Furthermore, this study used Huber’s six dimensions as an operationalization for the broader perspectives on health, as it is a broad definition covering most new definitions. Yet, other definitions and subscales, such as those available with the Impact on Participation and Autonomy survey, are of interest to further study. Similarly, experts did not always agree on item classification, which could indicate items were excluded that covered multiple dimensions as they could not simply be assigned to a single dimension. The classification of such items might have required the addition of an ‘overall’ dimension. Finally, future research should further study the potential use of the survey. For researchers and professionals the specificity of insight into the different dimensions of health in a population will be valuable when designing targeted interventions. Therefore, strong separation between dimensions should be a priority when aiming to tailor the survey to measure a broader concept of health at a population level. This study focuses on the performance of the survey in a Dutch population and for international use, the survey necessarily needs to be translated and retested. Additionally, testing of a more differentiating survey could determine whether creating distinction between these new health dimensions in populations can be attained quantitatively.

## Conclusion

The MyQualityOfLife.nl survey can be used to assess the broader concept of health in a population as well as the more specific ‘quality of life’ and ‘daily functioning’. This makes the survey a useful instrument for the evaluation of population health by new integrated care initiatives, providing them with the possibility to include this perspective in their approach. However, the survey lacks the ability to evaluate several of the new positive health dimensions separately. Further research is needed to determine whether this is due to the instrument or the positive health concept dimensions.

## Additional Files

The additional files for this article can be found as follows:

10.5334/ijic.3967.s1 Appendix 1. Positive Health infographic.

10.5334/ijic.3967.s2 Appendix 2. Results of response analyses of MKVL survey.

10.5334/ijic.3967.s3 Appendix 3. Exploratory Factor Analyses.

10.5334/ijic.3967.s4 Appendix 4. Reliability analyses.
