# Supplementary material for: Measuring Population Health from a Broader Perspective: Assessing the My Quality of Life Questionnaire
Source: Int J Integr Care. 2019 May 13;19(2):7. doi: 10.5334/ijic.3967 (PMC6524552; doi:10.5334/ijic.3967)
Supplement: Appendix 1. — Positive Health infographic. [file ijic-19-2-3967-s1.pdf]

Appendix 1. Positive Health infographic

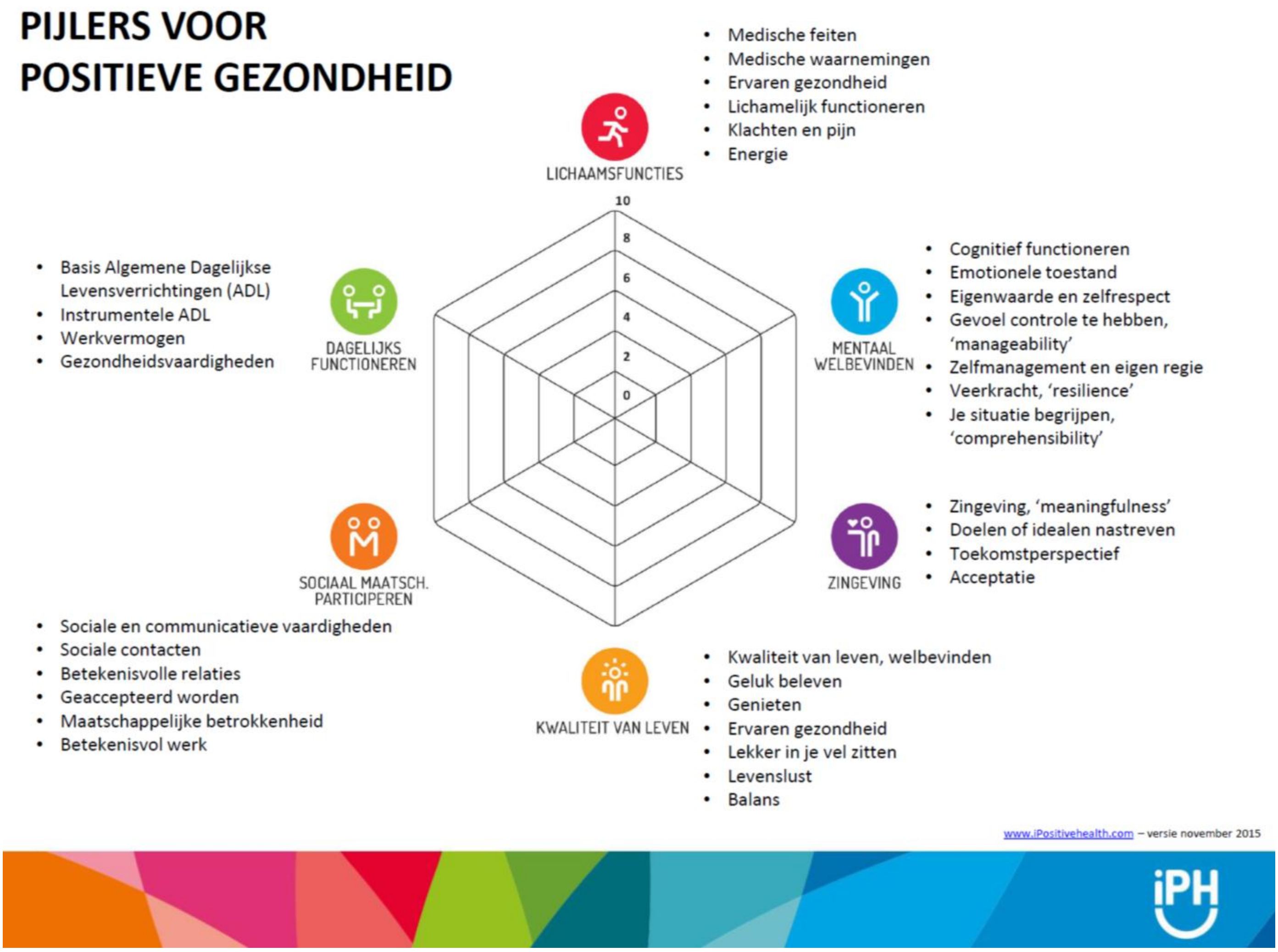

Figure A1.1. Scoring instrument Positive Health version October 2015 (language: Dutch)
