# Supplementary material for: Measuring Population Health from a Broader Perspective: Assessing the My Quality of Life Questionnaire
Source: Int J Integr Care. 2019 May 13;19(2):7. doi: 10.5334/ijic.3967 (PMC6524552; doi:10.5334/ijic.3967)
Supplement: Appendix 2. — Results of response analyses of MKVL survey. [file ijic-19-2-3967-s2.pdf]

Appendix 2. Results of response analyses of MKVL survey

Table A2.1. Response distributions (1/3)

	Never	Sometimes	Regularly	Most of the time	Always
**Quality of Life**					
I feel happy	2.2	23.4	23.6	43.6	7.2
I enjoy my life	2.2	22.6	23.7	39.3	12.1
I am happy with my life as it is	7.2	26.0	20.9	35.8	10.0
I think my life is meaningful and purposeful	5.5	21.4	19.5	37.1	16.5
**Life and living together**					
I live in a way that fits me	4.4	15.0	18.1	44.5	18.1
I make my own choices	0.8	8.6	15.8	48.2	24.1
I know what I want in life	1.6	10.3	15.8	48.2	24.1
I feel strong due to my life conceptions and beliefs	12.3	15.5	18.3	34.2	19.7
I feel good about the responsibilities I have	2.5	12.9	18.9	44.7	21.1
I have a good balance between activity and relaxation	8.2	24.9	24.3	32.7	9.8
I spent time on things I think are important	1.2	12.1	24.4	43.5	18.8
I have a good balance between being alone and among people	4.7	17.2	20.6	40.8	16.7
I have a warm and trusted relations with other people	2.2	14.3	19.9	41.8	21.8
I feel taken serious by other people	2.0	13.9	19.9	49.4	14.8
I feel accepted in my neighborhood/environment	2.7	11.3	16.5	43.8	25.7
I feel safe in my neighborhood/environment	2.0	7.8	14.9	44.7	30.5
I can go to others if I need help	3.4	17.1	17.1	34.6	27.7
I can mean something for other people	2.2	15.2	20.7	42.6	19.4
I feel useful	4.7	18.9	21.8	39.4	15.2
I dare to ask when I need help	4.3	26.8	19.3	31.2	18.5
**My health**					
I feel physically healthy	17.4	24.0	19.9	32.1	6.7
I feel mentally healthy	4.4	13.7	14.7	38.3	28.9
I feel fit enough to do what I want	14.3	27.0	19.6	28.5	10.7
I can deal with change and setbacks	3.3	18.5	18.2	45.2	14.8

Table A2.2. Response distribution (2/3)

	Very good	Good	Reasonably	Moderate	Bad
**Mobility**					
Go where I want in my residence, goes	56.0	27.9	11.0	3.5	1.6
Go when I want in my residence, goes	55.6	27.9	11.3	3.7	1.5
Visiting neighbors, friends and acquaintances when I want goes	44.9	26.3	14.3	8.4	5.5
Undertaking trips as I want to goes	36.3	20.2	15.6	13.1	14.8
Going to work and/or locations like I want goes	40.3	23.1	15.8	10.7	10.2

**Occupations and responsibilities in and around the house**					
The possibility to fill the role that fits me is	38.3	35.1	16.0	7.1	3.4
My contributions around the house as I want is	31.4	29.3	19.7	12.7	7.0
Doing light household chores like I want is	34.3	34.2	18.0	9.1	4.3
Doing heavy household chores like I want is	28.2	27.9	18.3	12.7	13.0
Doing household chores when I want is	30.4	30.9	19.5	11.6	7.6
Doing chores around the house and in the garden like I want is	26.1	26.4	20.1	14.7	12.7
**Social contacts and relations**					
The possibility for an equivalent conversation with people I love is	43.0	39.3	11.0	4.6	2.1
The social contact with people I love is	44.0	40.6	9.9	4.0	1.5
The respect I receive from people I love is	40.2	44.2	10.7	3.4	1.5
The social contact with people I know less is	14.4	50.7	23.5	8.3	3.1
The respect I receive from people I know less is	14.1	51.8	23.3	8.0	2.8
The possibility to give and receive love and affection is	28.5	43.6	16.3	7.6	4.0
The possibility to see people as often as I want	20.4	34.2	22.5	14.6	8.3
The possibility to help or support people that need me is	20.9	38.6	21.7	11.8	6.9
**Personal care**					
Washing, dressing and grooming the way I want goes	53.1	30.7	10.1	3.9	2.2
Washing, dressing and grooming when I want goes	53.0	30.7	10.1	4.1	2.1
Going to bed and getting out of bed when I want goes	51.4	31.7	10.5	4.3	2.1
Going to the toilet when I need and want to goes	56.7	34.1	6.2	1.9	1.1
Eating and drinking when I want goes	55.9	34.8	6.5	1.9	0.9
Eating and drinking what I want goes	53.0	33.3	8.8	3.3	1.6
Eating and drinking where I want	51.8	31.9	9.3	4.4	2.7
**Work and education**					
The possibility to do paid or unpaid work is (NA = 50.4%)	10.0	17.2	8.5	5.5	8.5
The possibility to do work that fits me is (NA = 47.5%)	11.2	20.2	9.5	5.0	6.7
Contact with my colleagues is (NA = 46.5%)	14.3	28.1	6.7	2.7	1.6
The possibility to improve or keep my position is (NA = 54.1%)	7.7	17.3	9.2	5.3	6.5
The possibility to change position or employer is (NA = 64.2%)	3.8	7.6	6.8	7.0	10.6
The possibility to follow education of	6.3	12.4	8.2	5.9	7.8

my choice is (NA = 59.4%)					
**Money and time spent**					
The possibility to pay what I need with my income is	20.7	35.2	21.8	12.7	9.6
Keeping track of my money is	37.2	40.2	13.8	5.1	3.7
Keeping track of my expenses is	36.0	42.1	14.1	4.7	3.2
The possibility to spent my money as I want is	25.3	37.1	19.2	10.7	7.8
The possibility to spent my time as I want is	24.8	42.4	20.6	8.3	3.8
The possibility to spent my free time as I want is	24.7	39.7	19.9	10.3	5.6

Table A2.3. Response distributions (3/3)

	**1**	**2**	**3**	**4**	**5**	**6**	**7**	**8**	**9**	**10**
My life	0.7	0.9	2.2	3.9	7.7	14.0	26.1	32.9	9.4	2.2
My health	1.0	2.0	4.7	8.3	14.0	19.3	23.3	19.2	6.5	1.7
My possibility to live in a way that fits me	1.7	2.2	3.9	6.1	11.3	15.9	21.9	24.3	9.3	3.4
My aids (NA = 52.1%)	2.9	1.4	1.8	2.7	6.4	8.7	10.9	9.4	2.3	1.3
Help from people around me (NA = 34.0%)	4.0	2.5	2.8	3.4	5.7	8.3	13.7	16.4	5.7	3.5
Help from professionals (NA = 50.0%)	5.6	2.0	2.7	3.0	5.5	7.3	10.1	9.9	2.8	1.3
My neighborhood/environment	8.8	3.2	4.0	4.4	9.0	13.7	22.9	23.5	6.4	4.0
Contact with municipalities if I need help (NA = 51.7%)	7.0	2.6	3.5	3.8	6.8	8.2	8.5	5.7	1.3	0.8
Contact with my insurance company if I need medical help (NA = 36.8%)	3.5	1.8	2.3	2.7	6.1	10.0	16.1	14.6	4.0	2.1
My faith in the future if I think about my own life	3.3	2.8	4.4	5.9	10.8	15.0	22.4	23.7	8.0	3.6
My faith that care will remain affordable for everyone in the Netherlands	16.8	9.1	11.9	13.3	17.3	15.0	9.2	5.0	1.3	1.1
