# Supplementary material for: Measuring Population Health from a Broader Perspective: Assessing the My Quality of Life Questionnaire
Source: Int J Integr Care. 2019 May 13;19(2):7. doi: 10.5334/ijic.3967 (PMC6524552; doi:10.5334/ijic.3967)
Supplement: Appendix 3. — Exploratory Factor Analyses. [file ijic-19-2-3967-s3.pdf]

Appendix 3. Exploratory Factor Analyses

Table A3.1. Pattern Matrix of Exploratory Factor Analyses

**Factor**	**1**	**2**
Are you going to fill out the survey yourself of do you receive help?	0.041	0.123
I feel happy	**-0.787**	0.142
I enjoy my life	**-0.806**	0.136
I am happy with my life as it is	**-0.774**	0.084
I think my life is meaningful and purposeful	**-0.792**	0.144
I live in a way that fits me	**-0.717**	0.044
I make my own choices	**-0.611**	0.034
I know what I want in life	-0.595	0.207
I feel strong due to my life conceptions and beliefs	-0.552	0.149
I feel good about the responsibilities I have	**-0.723**	0.139
I have a good balance between activity and relaxation	**-0.689**	0.067
I spent time on things I think are important	**-0.676**	0.144
I have a good balance between being alone and among people	**-0.707**	0.114
I have a warm and trusted relations with other people	**-0.721**	0.162
I feel taken serious by other people	**-0.739**	0.108
I feel accepted in my neighborhood/environment	**-0.712**	0.129
I feel safe in my neighborhood/environment	**-0.603**	0.059
I can go to others if I need help	**-0.709**	0.111
I can mean something for other people	**-0.606**	0.039
I feel useful	**-0.764**	0.092
I dare to ask when I need help	**-0.636**	0.112
I feel physically healthy	-0.469	-0.241
I feel mentally healthy	**-0.752**	0.140
I feel fit enough to do what I want	-0.557	-0.202
I can deal with change and setbacks	**-0.679**	0.101
I do not use aids	0.055	-0.335
Receive help from: my partner or children at home	-0.100	0.260
Receive help from: other family	-0.102	0.240
Receive help from: friends or acquintances	-0.156	0.261
Receive help from: neighbours or neighbourhoodactivities	-0.156	0.262
Receive help from: volunteers and voluntaryservices	-0.087	0.279
Receive help from: peers	-0.029	0.232
Transportationservices	-0.045	0.392
Domestic help (private)	-0.135	0.232
Domestic help (home care)	-0.028	0.332
Personal care	-0.099	0.427
Go where I want in my residence, goes	0.206	**0.637**
Go when I want in my residence, goes	0.219	**0.647**

Visiting neighbors, friends and acquaintances when I want goes	0.372	0.578
Undertaking trips as I want to goes	0.366	0.568
Going to work and/or locations like I want goes	0.338	**0.624**
The possibility to fill the role that fits me is	0.430	0.508
My contributions around the house as I want is	0.352	**0.608**
Doing light household chores like I want is	0.351	0.593
Doing heavy household chores like I want is	0.304	0.568
Doing household chores when I want is	0.346	0.573
Doing chores around the house and in the garden like I want is	0.332	0.571
The possibility for an equivalent conversation with people I love is	0.572	0.197
The social contact with people I love is	0.595	0.182
The respect I receive from people I love is	0.570	0.140
The social contact with people I know less is	0.585	0.192
The respect I receive from people I know less is	0.564	0.203
The possibility to give and receive love and affection is	**0.614**	0.161
The possibility to see people as often as I want	0.550	0.361
The possibility to help or support people that need me is	0.482	0.387
Washing, dressing and grooming the way I want goes	0.238	**0.690**
Washing, dressing and grooming when I want goes	0.253	**0.693**
Going to bed and getting out of bed when I want goes	0.304	0.597
Going to the toilet when I need and want to goes	0.220	**0.636**
Eating and drinking when I want goes	0.325	0.598
Eating and drinking what I want goes	0.331	0.570
Eating and drinking where I want	0.318	**0.615**
Yes, I have a paid job.	-0.103	-0.236
The possibility to do paid or unpaid work is	0.388	0.275
The possibility to do work that fits me is	0.451	0.288
Contact with my colleagues is	0.482	0.172
The possibility to improve or keep my position is	0.514	0.215
The possibility to change position or employer is	0.415	0.217
The possibility to follow education of my choice is	0.453	0.261
The possibility to pay what I need with my income is	0.404	0.243
Keeping track of my money is	0.393	0.207
Keeping track of my expenses is	0.393	0.161
The possibility to spent my money as I want is	0.442	0.210
The possibility to spent my time as I want is	0.484	0.262
The possibility to spent my free time as I want is	0.514	0.269
The feeling that dominates when you think about the coming six months	-0.160	0.003
My life	**-0.732**	-0.011
My health	-0.485	-0.283
My possibility to live in a way that fits me	**-0.645**	-0.148

My aids	-0.384	-0.048
Help from people around me	-0.533	0.028
Help from professionals	-0.486	-0.019
My neighborhood/environment	**-0.675**	-0.061
