# Supplementary material for: Measuring Population Health from a Broader Perspective: Assessing the My Quality of Life Questionnaire
Source: Int J Integr Care. 2019 May 13;19(2):7. doi: 10.5334/ijic.3967 (PMC6524552; doi:10.5334/ijic.3967)
Supplement: Appendix 4. — Reliability analyses. [file ijic-19-2-3967-s4.pdf]

Appendix 4. Reliability analyses

Table A4.1. Results of reliability analyses factor 1 (Cronbach's Alpha = 0.779)

Overall Cronbach's Alpha		0.941	0.953
	Corrected Item-Total Correlation	Cronbach's Alpha if Item Deleted	Cronbach's Alpha if Item Deleted
I feel happy	0,77	0,937	0,95
I enjoy my life	0,798	0,937	0,949
I am happy with my life as it is	0,772	0,937	0,949
I think my life is meaningful and purposeful	0,77	0,937	0,949
I live in a way that fits me	0,701	0,938	0,95
I make my own choices	0,557	0,94	0,952
3e. Stelling Ik voel me goed bij de verantwoordelijkheden die ik draag	0,7	0,938	0,95
I feel good about the responsibilities I have	0,717	0,938	0,95
I have a good balance between activity and relaxation	0,664	0,939	0,951
I spent time on things I think are important	0,697	0,938	0,95
I have a good balance between being alone and among people	0,632	0,939	0,951
I have a warm and trusted relations with other people	0,667	0,939	0,951
I feel taken serious by other people	0,639	0,939	0,951
I feel accepted in my neighborhood/environment	0,551	0,94	0,952
I can go to others if I need help	0,625	0,939	0,951
I can mean something for other people	0,607	0,939	0,951
I feel useful	0,741	0,937	0,95
I dare to ask when I need help	0,56	0,94	0,952
I feel mentally healthy	0,692	0,938	0,95
I can deal with change and setbacks	0,642	0,939	0,951
~~The possibility to give and receive love and affection is~~	-0,607	0,953	excluded
My life	0,771	0,937	0,949
My possibility to live in a way that fits me	0,705	0,939	0,951
My faith in the future if I think about my own life	0,713	0,939	0,952

Table A4.2. Results of reliability analyses factor 1 (Cronbach's Alpha = 0.944)

Overall Cronbach's Alpha		0.900	0.931
	Corrected Item-Total Correlation	Cronbach's Alpha if Item Deleted	Cronbach's Alpha if Item Deleted
~~I do not use aids~~	-0.373	0.931	excluded
Go where I want in my residence, goes	0.779	0.881	0.921
Go when I want in my residence, goes	0.795	0.879	0.920
Going to work and/or locations like I want goes	0.743	0.886	0.927
My contributions around the house as I want is	0.725	0.885	0.926
Washing, dressing and grooming the way I want goes	0.827	0.877	0.917
Washing, dressing and grooming when I want goes	0.834	0.876	0.917
Going to the toilet when I need and want to goes	0.744	0.885	0.925
Eating and drinking where I want	0.738	0.883	0.923
